# Childbirth and consequent atopic disease: emerging evidence on epigenetic effects based on the hygiene and EPIIC hypotheses

**DOI:** 10.1186/s12884-015-0768-9

**Published:** 2016-01-13

**Authors:** H. G. Dahlen, S. Downe, M. L. Wright, H. P. Kennedy, J. Y. Taylor

**Affiliations:** School of Nursing and Midwifery, Western Sydney University, Locked Bag 1797, Penrith, 2751 NSW Australia; University of Central Lancashire, Preston, PR3 2LE Lancashire UK; Yale School of Nursing, 400 West Campus Drive, West Haven, CT 06516 USA

**Keywords:** Epigenetics, Birth, Caesarean section, Syntocinon, Microbiome, Antibiotics

## Abstract

**Background:**

In most high and middle income countries across the world, at least 1:4 women give birth by cesarean section. Rates of labour induction and augmentation are rising steeply; and in some countries up to 50 % of laboring women and newborns are given antibiotics. Governments and international agencies are increasingly concerned about the clinical, economic and psychosocial effects of these interventions.

**Discussion:**

There is emerging evidence that certain intrapartum and early neonatal interventions might affect the neonatal immune response in the longer term, and perhaps trans-generationally. Two theories lead the debate in this area. Those aligned with the hygiene (or ‘Old Friends’) hypothesis have examined the effect of gut microbiome colonization secondary to mode of birth and intrapartum/neonatal pharmacological interventions on immune response and epigenetic phenomena. Those working with the EPIIC (Epigenetic Impact of Childbirth) hypothesis are concerned with the effects of eustress and dys-stress on the epigenome, secondary to mode of birth and labour interventions.

**Summary:**

This paper examines the current and emerging findings relating to childbirth and atopic/autoimmune disease from the perspective of both theories, and proposes an alliance of research effort. This is likely to accelerate the discovery of important findings arising from both approaches, and to maximize the timely understanding of the longer-term consequences of childbirth practices.

## Background

For most of human history birth has been seen as a social event occurring at home amongst family and friends with minimal intervention [1, 2]. However, childbirth has also been seen as dangerous for both mother and baby [3]. Each year 289,000 women and 2.9 million babies die in the first month of life, with most of these deaths occurring in Africa and India [4]. The almost universal response to this level of risk has been a movement of birth into institutional settings, to be closer to potentially lifesaving skills, interventions, and treatments. Maternal mortality and morbidity does tend to drop when birth is institutionalized, although there are debates as to the aetiology of this effect [5]. Some argue that the association is not causal, as it often coincides with improvement in social and economic conditions, and with the provision of specific drugs such as antibiotics and treatments for postpartum hemorrhage [6].

More recently, concern has been expressed that the pendulum has swung excessively towards prophylactic and unnecessary interventions for childbearing women and babies - ‘just in case’ [7]. In most high and middle income countries across the world, more than 1:4 women now give birth by cesarean [8]. Even in low income countries, some (wealthier) women are subject to very high rates of cesarean section [9]. Rates of labour induction and augmentation are rising steeply, and up to 50 % of laboring women and newborns in specific settings receive antibiotics [10]. Governments and international agencies are increasingly concerned about the clinical, economic and psychosocial effects of these interventions [11, 12]. The recent Lancet Series on Midwifery has noted the need for balance between excessive levels of intervention (and associated morbidity) and lack of appropriate intervention when it is needed [13].

More recently some researchers have begun to examine the rapid rise in intrapartum interventions such as caesarean sections [14, 15], the use of exogenous oxytocin for labour induction and/or augmentation [16], the use of intrapartum and neonatal antibiotics [10], and rates of infant formula feeding [17] as possibly associated with a similar steep rise in reports of asthma and atopic disorders globally [18, 19].

Two theories lead the debate in this area. Those aligned with the hygiene (or ‘Old Friends’) hypothesis have examined the effect of gut microbiome colonization secondary to mode of birth and intrapartum/neonatal pharmacological interventions on immune response and epigenetic phenomenon [20, 21]. Those working with the EPIIC (Epigenetic Impact of Childbirth) hypothesis are concerned with the effects of eustress and dys-stress on the epigenome, secondary to mode of birth and labour interventions [19]. This paper describes key epigenetic concepts, followed by a comparative presentation of the two theoretical propositions for potential causative associations between birth related events and atopic disease. It then proposes that those working from the perspective of both these theories might unite their research efforts to generate a more effective synergistic analysis of the situation.

## Discussion

### Epigenetics

Each body cell has the same DNA and genes, but their expression can vary [22]. Epigenetic regulation serves as a precise process that regulates the expression of certain genes and the silencing of others. Epigenetics is a rapidly developing field in biomedicine and is important for understanding the course of the human lifecycle and how resistance and susceptibility to disease develop. It is also an emerging field in nursing and midwifery science [23] with focus on topics such as hypertension and preeclampsia [24], renal disease [25] and other chronic conditions [19]. The examination of mode of birth, atopic disease, and epigenetics are timely and important, since it has been hypothesized that they may be interrelated.

DNA methylation is a specific epigenetic alteration in which a methyl group attaches to a cytosine (C) residue in DNA, which is followed by a guanine (G) residue connected by a phosphate bond, commonly referred to as a CpG dinucleotide. Between 60 and 90 % of cytosines are methylated in human DNA [26] and the establishment of “normal” DNA methylation patterns are necessary for embryonic development. DNA methylation occurs mainly at the C5 position of CpG dinucleotides. Normal patterns of methylation are required for the differentiation of cell types. For example, every cell in an individual’s body has the same DNA sequence, and methylation patterns present on the DNA sequence are specific to tissue type [27–29]. In other words, the methylation pattern for a cell in the heart will be different from the methylation pattern on a cell in the eye, even though the DNA sequence is exactly the same. Alterations in methylation patterns also explain some of the processes that occur in complex disease states, such as delayed onset disease or situations where only one identical twin develops cancer [30–33].

DNA methylation patterns are known modulators of immune function and alterations have been associated with processes that moderate immune development. Examples of these are listed in Table 1. Martino et al. [34] found that exposures within the first years of life alter methylation patterns on monoculear cells. They identified significant methylation changes in cell signaling and communication pathways associated with immune response and development. They found that the methylation associated with immune function in fetal cells were substantially different relative to the first 5 years of life and underwent the most significant changes in the first year of life. They suggest this may be related to the lack of antigenic challenges to the fetal immune system in utero. Because the methylation related to programming the immune system appears to be specifically susceptible to alterations early in life, exposures occurring during the labor and birth process should be carefully considered. Additionally, methylation alterations in various cell types of the immune system contribute to how the body responds to antigenic challenges throughout life. For example, T-lymphocyte cell function and cytokine expression are altered by methylation patterns present on T cells [35] and DNA methylation patterns are altered by bacterial infection [36, 37]. Different methylation patterns associated with the development of disease have been identified in animal models when they are colonized with altered bacterial populations after birth [38]. Since DNA methylation vitally contributes to cell differentiation in the immune system and programming memory in immune cells, it is an excellent candidate for identifying unknown mechanisms that may be associated with altered immune function, such as asthma and atopic disease.

**Table 1:** DNA Methylation and immune function

Immune function or Alteration	Reference
Bacterial infection induces hypermethylation	[37]
Discriminates between regulatory and conventional T cells	[75]
Downregulation of IFN-γ in fetus, helps prevent fetal loss	[76]
Early-life stress alters T-cell methylation	[69]
IgE production	[77]
Immune cell signaling and communication pathways	[34]
Maintenance of T cell memory and cytokine expression pattern	[35]
Maternal bacterial infection promotes fetal hypermethylation	[78]
Number and function of regulatory T cells	[79]
Viral infection increases DNA methylation	[36]

### The hygiene (‘Old Friends’) hypothesis

Supporters of the ‘hygiene hypothesis’ have proposed that over the past century declining family size, improved household amenities, and higher standards of personal cleanliness have reduced opportunities for cross infection in young families leading to more widespread expression of allergy responses. Strachan [39] was one of the first people to publish on the hygiene hypothesis and this has remained a dominant explanation for the increase in atopic and autoimmune diseases we see today. However, Strachan himself has recently questioned aspects of this theory [40] as have other researchers working in this area [41, 42]. Rook and colleagues have proposed that the regulatory capacity of the gut microbiotia is primed by so called ‘Old Friends’ [43]. These are microbes that formed part of human evolution, from Neolithic times, and that are still present in the human gut. The theory proposes that, without the presence of these microbes, the human immune system will not develop and act effectively when it encounters potential antigenic stimulants. Even more recently, the paediatric community has begun to critique the original hygiene hypothesis – for instance Weiss [42] notes that, although diseases such as eczema, asthma, allergic rhinoconjunctivitis, inflammatory bowel disease, and diabetes are rising significantly in high income nations worldwide, changes in hygiene practices preceded the epidemic of these diseases by many years.

These, and similar critiques, have led to what has been termed the ‘extended hygiene hypothesis’ suggesting that, rather than waiting for exposure to bacteria to prime the immune system, in-utero, and in the first hours, weeks or months of life, the baby needs to gather a community of pre-existing microbes that come from the mother, as well as from the surrounding environment. Establishing the gut microbiota (which is increasingly being described as an active organism rather than a passive colonisation) may be important in protecting the child and later on the adult against atopic and immunological diseases [44]. Disturbances in this process could be linked to developing infectious, inflammatory, and allergic diseases later in life [44, 45]. There is also emerging evidence of the importance of the gut bacteria modulating the blood brain barrier [46].

The extended hygiene hypothesis may be supported by evidence that babies born by caesarean delivery have different gut microbiota in the first hours and days of life to those born vaginally, suggesting that the route of birth may be fundamental to the physiology of the gut flora. The recent CHILD study from Canada used DNA sequencing to detect microbes in faecal samples from infants at age four months. They found infants born by caesarean delivery have low bacterial richness and diversity compared to those born vaginally [20]. There is mounting evidence that the development of IgE-mediated sensitization to food allergens is higher in children born by caesarean delivery [47].

There is also evidence to indicate a marked increase in children’s susceptibility to a range of immune related disorders if they are born by caesarean delivery, and particularly when it is performed electively without concurrent labour [15]. Following cesarean delivery there are higher numbers of cells secreting immunoglobulins (IgA and IgG) at 1 year of age, and some studies have found increased rates of asthma, gastroenteritis, rhinitis, food allergies, and type 1 diabetes [15, 48, 49]. Other studies have produced conflicting findings, particularly with respect to inhalant atopy or atopic dermatitis [50]. These authors hypothesise that the effect of caesarean section may not contribute significantly to the observed rise in allergic disease.

#### DNA methylation and altered gut colonization

Alterations in DNA methylation patterns related to altered initial colonization of the gut have been identified in animal models. Back in 1915, Kendall determined that microbes, which colonize the gastrointestinal tract at birth, were involved in normal development of the immune system [50]. However, the biological mechanism of how this occurred remained unknown for years. Olszak et al. [38] hypothesized that the type of bacteria that colonize the gastrointestinal tract in the neonatal period, has an effect on the function of cells in the immune system. The study was performed on germ-free and specific-pathogen free mice. Olszak’s study also suggested that microbial exposure alters gene expression in specific tissues. They found that hypermethylation of CpG sites in colon and lung tissues of the chemokine (C-X-C motif) ligand 16 (*CXCL16*) gene occurred when specific pathogens were not present during early life development. The *CXCL16* gene encodes a chemokine receptor on invariant natural killer T cells (iNKT), resulting in higher accumulations of iNKT cells that are involved with inflammatory processes. Thus higher accumulation of the iNKTs only occurred in the colon and lung when specific bacteria were not present. The authors hypothesized an environmental exposure later in life triggers various inflammatory disease processes programmed by the altered methylation in the bowel and lungs, such as asthma and irritable bowel syndrome. Therefore, exposure to bacteria early in development affects the programming of the immune system, in mice, by causing perturbation in DNA methylation patterns. They concluded the findings could be extrapolated to humans because the mouse model used is similar to human cells. Further studies investigating alterations of methylation patterns in humans related to the effects of antibiotics and subsequent alteration in the microbiome could identify mechanisms of altered immune programming, and this may begin at the point of entry at birth. These findings have relevance to early infant development and mode of feeding.

#### Mode of birth and breastfeeding

Mode of birth (cesarean or vaginal) and whether or not the infant breastfeeds are also being studied as possible determinants of gut microbiota development [51, 52]. Caesarean delivery prevents normal colonisation in the baby’s gut, because of lack of exposure to the mother’s microbes. Breastfeeding is thought to lead to healthy gut microbiota by providing selective metabolic substrates for ‘good’ bacteria to exist [20, 52].

#### Antibiotic use

To date, primary research outcomes for studies investigating intrapartum antibiotic administration have been related to maternal illness and health outcomes [53]. Intrapartum antibiotic administration prior to initiating the incision when cesarean deliveries are performed significantly decreases infectious morbidity outcomes in women [54]. Ledger and Blaser pose that U.S. physicians prescribe too many antibiotics for the pregnant woman and fetus in utero [10]. They cited practice modifications to prevent group B streptococcus (GBS) infections in newborn and postpartum maternal infection after caesarean delivery has led to the use of pre-delivery antibiotics of ≥40 % of women in labour, which cross to the fetus. However, there has been little research regarding the long-term effects of antepartum or intrapartum antibiotic use on the infant. In their review of 86 studies, Smaill and Gyte [53] noted the effects of intrapartum antibiotic administration on the infant are largely unknown, limiting the ability to accurately evaluate the potential benefit and/or harm of intrapartum administration. A 2009 Cochrane Review reported that the recommendations for neonatal GBS antibiotic prophylaxis are based on flawed studies, and that, due to current clinical protocols, it is unlikely unbiased studies will be initiated [55]. The incidence of neonatal GBS sepsis during the first 7 days of life has declined 80 % (though the same cannot be said for late onset GBS) since implementation of prophylactic antibiotic administration in women colonized with GBS, suggesting a straightforward recommendation for routine prophylactic treatment, at first glance [56]. However, despite the methodological and ethical difficulties, future studies are needed to weigh the risks and harms of antibiotic exposure during birth. This is increasingly apparent as evidence accumulates around the potential risks of routine prophylactic use of antibiotics, including increased rates of asthma in early childhood associated with maternal antibiotic use, which suggests a role for bacterial ecology in the perinatal period [57, 58]. There are also studies showing associations between maternal antibiotic use before and during pregnancy and infant allergy to cow’s milk [59]; and that administration of antibiotics to children under the age of 6 months is linked with higher rates of obesity for children of parents at normal weight, independent of mode of birth [60]. Epidemiological research has also shown an association between exposure to antibiotics during pregnancy and infant birth weight, and epigenetic analysis has demonstrated altered DNA methylation in these circumstances, which has been associated with obesity as well as other disorders later on in life [21]. Antibiotics have been shown to trigger altered gene expression and changes in gut microbes in rats. This has been found to influence immune system development and function [61, 62]. Fetal and/or neonatal exposure to antibiotics may lead to a dysfunctional development of the immune system initiating a cascade of events associated with future health conditions. Csoka and Szyf [63] hypothesized that pharmaceuticals create a gene-environment interaction which prompts cells to adapt through methylation and that such epigenetic changes may persist after the drug has ceased. As an alternative explanation, a recent study has suggested that maternal antibiotic use may be a surrogate marker of a mother’s general propensity for infections, and that this might be the underlying link between a mothers use of antibiotics and the risk of asthma in the offspring [64]. This indicates the aetiology is complex and confounded by other pregnancy and intrapartum practices. Long term studies are needed to determine the aetiology and inter-generational outcomes of the current high rates of antibiotic administration during labour and birth [10].

### The EPIIC hypothesis

The EPIIC hypothesis published in 2013 proposes that, when labor and birth occurs primarily without intervention (when the process proceeds physiologically) a healthy positive form of stress (eustress) is exerted on the fetus [19]. This then has physiological epigenomic effect on specific genes, especially those that program immune responses, genes responsible for weight regulation, and specific tumor-suppressor genes. There is biological evidence for an altered stress (dys-stress) response in babies born after either elective cesarean section (when salivary cortisol response to stress seems to be down regulated at least for the first 2 months) or instrumental vaginal delivery (resulting in upregulation) when compared to those born after vaginal birth [65]. The EPIIC hypothesis suggests that the middle range of stress is physiologically optimal, and that this might be stimulated by non-pathological labour pain and the physical and psychological effort of labour. These processes are hypothesised to be essential both to enable the baby to be born, and as the stimulation for the optimal cortisol (stress) response to enable effective epigenetic priming. Excessively high levels (dys-tress) and excessively low levels (astress) are hypothesised to be associated with a-stress, both of which could be linked to pathological priming.

Others have shown differences in DNA methylation at the time of birth, by mode of birth [66, 67]. Very recently Almgre and colleagues [67] examined the cord blood of 27 babies born by caesarean section and 37 born vaginally. They found DNA from CD34+ cells from infants delivered by caesarean section were globally more methylated (+2 %) than DNA from infants delivered vaginally (*p* = 0.02). There was also a strong CpG-specific relationship between the duration of labour and locus specific DNA methlyation in a subset of identified genes. The authors hypothesize that altered methylation can create poised, replication-heritable epigenetic marks, not immediately influencing gene transcription until a second hit arrived causing disease. The authors concluded that mode of delivery affects the epigenetic state of neonatal hematopoietic stem cells and this may have implications for health and disease in later life. Interestingly the authors make the point that while the hygiene hypothesis postulates that it is the lack of colonization of the infant with maternal gut flora after caesarean that may prevent the initiation of the immune system of the newborn, they found a correlation between the duration of labour and DNA methylation, indicating that methylation status at the start of vaginal birth resembles that of caesarean born infants (unexposed to labour and ruptured membranes), after which DNA methylation differences progressively change along with delivery. They suggest this shows the findings are directly related to labour itself [67].

Use of intrapartum oxytocin is increasing, and this is important in terms of the influence of oxytocin on stress response and maternal-paternal-infant bonding in the first few days of life [68]. Altered methylation in T-cells has been observed in rhesus monkeys within 14 days after birth if offspring are immediately removed from their mothers and raised by a surrogate in a nursery [69]. Notably the changes in methylation persist throughout the lifespan suggesting alterations in early life can influence immune programming. Further Szyf, et al. [70] have examined the effects of altered timing of “normal stress” events, specifically maternal weaning. They found that DNA methylation patterns are altered for infants weaned early when compared to infants of a similar age who are not weaned, and that these patterns are similar to older infants who have been weaned at a developmentally appropriate time. Interestingly, the methylation patterns become divergent later in life between groups weaned early versus those weaned at an appropriate time. These data suggest that the timing of both eustress and dys-stress events may also be critically important for development, and that pathological early life programming may be compensated for in the short term, but that this compensatory mechanism might become overwhelmed over time.

Given the known link between the stress response, subsequent DNA methylation patterns and epigenetic changes that are associated with auto-immune response, [19] the EPIIC hypothesis suggests that (at least part of) the explanatory factor for the observed associations between mode of birth and use of intrapartum interventions and later chronic autoimmune disease in the infant is relative dys-stress (up or down regulation of cortisol, for instance) when compared to the eustress of physiological labour and birth. This is also an argument for the importance of the ‘labouring’ of labour, as a complex salutogenic phenomenon that is an essential, priming agent in the dynamic process of labour and birth [71]. Specifically, the EPIIC hypothesis proposes that reduced or elevated levels of cortisol, adrenalin, and oxytocin produced during labour may lead to fetal epigenomic remodelling anomalies which exert influence on abnormal gene expression. This reprogramming could manifest in a range of diseases and biobehavioral problems in the neonate and later on in the adult. This suggests that the physiology of labour and birth may be crucial to epigenetic remodelling, between fetal and extrauterine life [19].

Given that environmental exposures may alter DNA methylation patterns associated with phenotypic differences in adults, it is likely that preconception and antenatal exposures could influence the epigenetic signature of the developing fetus. The timing, duration, combination, and pattern of exposures play a critical role in contributing to the development of altered phenotypes [72]. In this debate, we aim to highlight elements that could contribute to the programming of immune dysfunction specifically related to events during intrapartum period. To date, evaluation of lasting effects of intrapartum maternal interventions on offspring health has been largely understudied. This is an area of inquiry that is in need of pursuit.

## Summary

### Uniting the extended hygiene hypothesis and EPIIC hypothesis

**Fig. 1:**
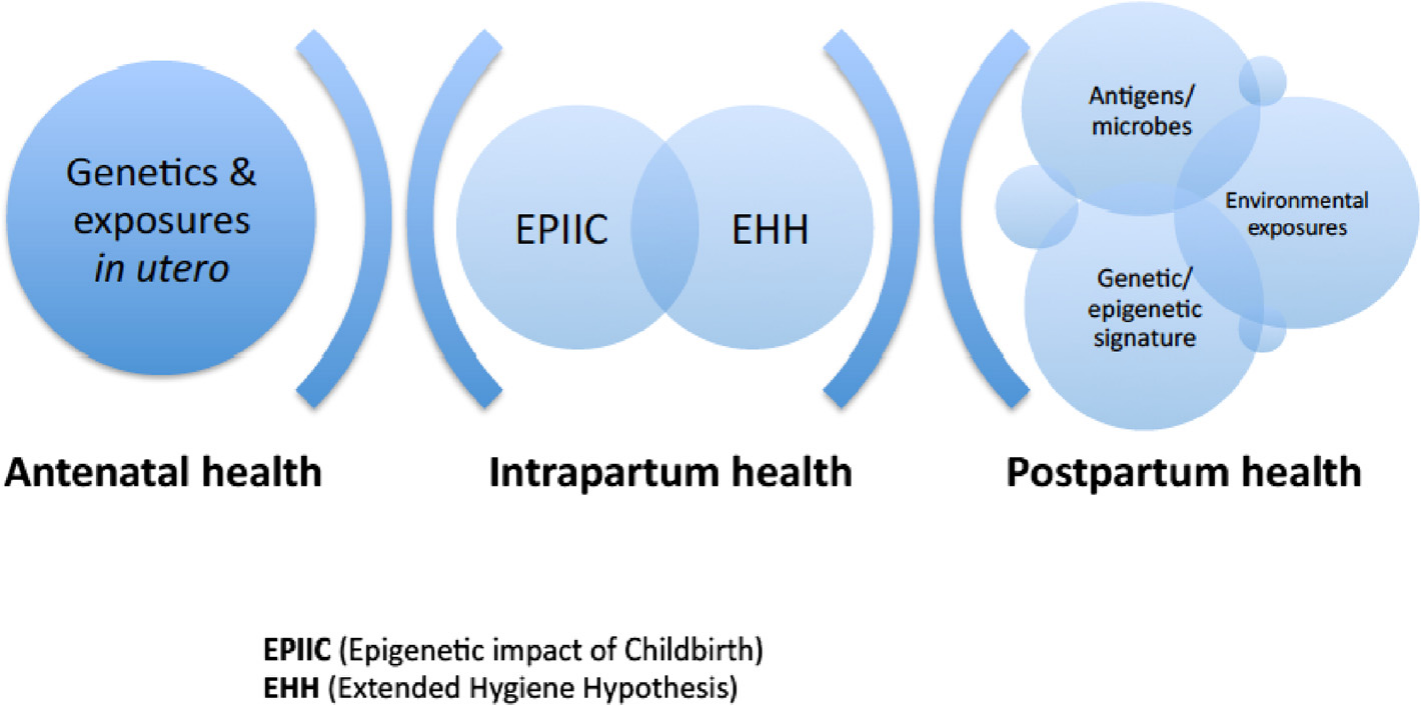
Additive influences on offspring health

Together or separately, the mounting evidence related to both the extended hygiene hypothesis (EHH) and the EPIIC hypothesis suggests that factors occurring during the intrapartum and early postnatal period may lead to fetal epigenomic remodeling anomalies, which exert influence on abnormal gene expression. These include mode of birth, dys-stress during labour and in the early postnatal period (a stress response that is either depressed below or elevated above a physiological eustress norm), and/or the effect of pharmacological agents such as antibiotics, or of mode of infant feeding (Fig. 1).

It is highly likely than more than one factor is behind the recent rise in atopic and autoimmune disease, and a combination of the extended hygiene hypothesis and EPIIC hypothesis may provide interesting answers in the future. In this paper we have further developed on the EPIIC hypothesis, which was published in 2013, to encourage researcher to consider the complexity and interconnectivity of this hypothesis with others such as the EHH. We propose an alliance of research effort. In the first instance, information is required about the baseline physiology of labour and birth. It is very unlikely that this can be done in hospital maternity wards, where most births take place, as the protocols and procedures and routine interventions used in maternity care in most hospitals across the world militate against physiological processes of birth. It is therefore vital to carefully examine the normal physiology of labour and birth in settings where women are undisturbed as far as possible. This is likely to be in birth centres, and at home births. Once this baseline is established, interventions in the process, such as caesarean section, use of forceps, induction of labour, augmentation with oxytocin and the introduction of antibiotics, should also be explored for their short- and long-term effects. Collaboration of scientists in these efforts is likely to accelerate the discovery of important findings arising from both approaches, and to maximize the timely understanding of the longer-term consequences of the way childbirth is currently managed in most countries of the world.

## Limitations and further discussion

Although this paper focuses on a specific hypothesis relating to epigenetic changes during childbirth and the possible consequent effects on immunity, we are aware that other mechanisms and health outcomes could be involved [23]. For example, in this paper we did not cover histone deacetylation nor the multiple other epigenetic mechanisms that may be associated with alterations in immune programming. Even within histone research, there are a range of modifications that could be operating (summolation, phosphorylation, methylation etc.). Chromatin remodeling or microRNA differences may also be involved. There has been a concentration of effort on understanding the DNA methylation of various diseases [24, 25], it is likely that methylation may be working in concert with other alterations or other environmental variables to result in various phenotypes. In this manuscript we do not intend to encompass all potential epigenetic alterations but to offer a hypothesis on how these alterations in methylation during the intrapartum period (a highly understudied but very important and vulnerable time) may affect immunity. We have also not examined the question of maternal stress during pregnancy and immunomodulation which may impact on the expression of allergic disease in children [73]. Indeed, there is evidence that maternal stress during pregnancy is associated with elevated cord blood IgE [74]. Our intention is not to propose that there is one simple answer to this complex clinical question. We do, however, believe that our EPIIC hypothesis breaks new ground in our cross-disciplinary focus on the potential trans-generational impact of the intrapartum period.

## Conclusion

In this paper we examine the current and emerging findings relating to childbirth and atopic/autoimmune disease from the perspective of both the Extended Hygiene Hypothesis (EHH) and the Epigentic Impact of Childbirth (EPIIC) hypothesis and we propose an alliance of research effort. This is likely to accelerate the discovery of important findings arising from both approaches, and to maximize the timely understanding of the longer-term consequences of childbirth practices.

## References

[1] Kitzinger S (2000). Rediscovering Birth.

[2] Jordan B (1993). Birth in Four Cultures: a cross cultural investigation of childbirth in Yucantan, Holland, Sweden and the United States.

[3] Murphy-Lawless J (1998). Reading Birth and Death: A History of Obstetric Thinking.

[4] Renfrew MJ, Homer CSE, Downe S, McFadden A, Muir N, Prentice T (2014). The Lancet's Series on Midwifery Executive Summary. Lancet..

[5] Van Lerberghe W, Matthews Z, Achadi E, Ancona C, Campbell J, Channon A, et al. Country experience with strengthening of health systems and deployment of midwives in countries with high maternal mortality. Lancet. 2014. 10.1016/S0140-6736(14)60919-3 24965819

[6] Loudon I (2000). Maternal mortality in the past and its relevance to developing countries today. Am. J. Clin. Nutr..

[7] Dahlen H, Tracy S, Tracy MB, Bisits A, Brown C, Thornton C (2014). Rates of obstetric intervention and associated perinatal mortality and morbidity among low-risk women giving birth in private and public hospitals in NSW (2000–2008): a linked data population-based cohort study. BMJ Open.

[8] Lavender T, Hofmeyr GJ, Neilson JP, Kingdon C, Gyte GML. Caesarean section for non-medical reasons at term. Cochrane Database of Systematic Reviews, 2012. ssue 3. Art. No.: CD004660. DOI:10.1002/14651858.CD004660.pub3. 10.1002/14651858.CD004660.pub2 16856054

[9] Sreevidya S, Sathiyasekaran BWC (2003). High caesarean rates in Madras (India): a population-based cross-sectional study. BJOG.

[10] Ledger WJ, Blaser MJ (2013). Are we using too many antibiotics during pregnancy?. BJOG.

[11] NSW Health (2010). Towards Normal Birth in NSW.

[12] Gibbons L, Belizán JM, Lauer JA, Betrán AP, Merialdi M, Althabe F (2010). The Global Numbers and Costs of Additionally Needed and Unnecessary Caesarean Sections Performed per Year: Overuse as a Barrier to Universal Coverage..

[13] ten Hoope-Bender P, de Bernis L, Campbell J, Downe S, Fauveau V, Fogstad H, et al. Improvement of maternal and newborn health through midwifery. Lancet. 2014. http://dx.doi.org/10.1016/S0140-6736(14)60930-2. 10.1016/S0140-6736(14)60930-2 24965818

[14] Bager P, Melbye M, Rostgaard K, Benn CS, Westergaard T (2003). Mode of delivery and risk of allergic rhinitis and asthma. J Allergy Clin Immunol..

[15] Hyde MJ, Mostyn A, Modi N, Kemp PR (2012). The health implications of birth by caesarean section. Biological Reviews.

[16] Buchanan SL, Patterson JA, Roberts CL, Morris JM, Ford JB (2012). Trends and morbidity associated with oxytocin use in labour in nulliparas at term. ANZJOG.

[17] McAndrew F, Thompson J, Fellows L, Large A, Speed M, Renfrew MJ. Infant feeding survey 2010. In. Edited by Health andSocial Care Information Centre; 2012.

[18] Wills-Karp M, Santeliz J, Karp CL (2001). The germless theory of allergic disease: revisiting the hygiene hypothesis. Nat Rev Immunol.

[19] Dahlen HG, Kennedy HP, Anderson CM, Bella F, Clark A, Foureur M (2013). The EPIIC hypothesis: Intrapartum effects on the neonatal epigenome and consequent health outcomes. Med Hypotheses.

[20] Azad MB, Konya T, Maughan H, Guttman DS, Field CJ, Chari RS, et al. Gut microbiota of healthy Canadian infants: profiles by mode of delivery and infant diet at 4 months. Canadian Medical Association 2013, CMAJ 2013. DOI:10.1503/cmaj.121189. 10.1503/cmaj.121189 PMC3602254 23401405

[21] Vidal AC, Murphy SK, Murtha AP, Schildkraut JM, Soubry A, Huang Z, et al. Associations between antibiotic exposure during pregnancy, birth weight and aberrant methylation at imprinted genes among offspring. Int J Obes (Lond). 2013;37:907–13. 10.1038/ijo.2013.47 PMC3705584 23609933

[22] Moshe E (2009). Early life, the epigenome, and human health. Acta Paediatr.

[23] Clark AE, Adamian M, Taylor JY (2013). An Overview of Epigenetics in Nursing. Nurs Clin N Am.

[24] Anderson CM, Ralph JL, Johnson L, Scheett A, Wright ML, Taylor JY (2015). First Trimester Vitamin D Status and Placental Epigenomics in Preeclampsia Among Northern Plains Primiparas. Life Sciences.

[25] Bomotti S, Smith JA, Zagel A, Taylor JY, Turner ST, Kardia SLR (2013). Epigenetic Markers of Renal Function in African Americans. Nurs Res Prac.

[26] Ehrlich M, Gama-Sosa MA, Huang LH, Midgett RM, Kuo KC, McCune RA (1982). Amount and distribution of 5-methylcytosine in human DNA from different types of tissues of cells. Nucleic Acids Res.

[27] Cedar H, Bergman Y (2012). Programming of DNA methylation patterns. Annu Rev Biochem.

[28] Jones PA (2012). Functions of DNA methylation: islands, start sites, gene bodies and beyond. Nat Rev Genet.

[29] Laird PW (2010). Principles and challenges of genomewide DNA methylation analysis. Nat Rev Genet.

[30] Boks MP, Derks EM, Weisenberger DJ, Strengman E, Janson E, Sommer IE (2009). The relationship of DNA methylation with age, gender and genotype in twins and healthy controls. PLoS One.

[31] Fraga MF, Ballestar E, Paz MF, Ropero S, Setien F, Ballestar ML (2005). Epigenetic differences arise during the lifetime of monozygotic twins. Proc Natl Acad Sci U S A.

[32] Kaminsky ZA, Tang T, Wang S-C, Ptak C, Oh GHT, Wong AHC (2009). DNA methylation profiles in monozygotic and dizygotic twins. Nat Genet.

[33] Petronis A (2001). Human morbid genetics revisited: relevance of epigenetics. Trends Genet.

[34] Martino DJ, Tulic MK, Gordon L, Hodder M, Richman TR, Metcalfe J (2011). Evidence for age-related and individual-specific changes in DNA methylation profile of mononuclear cells during early immune development in humans. Epigenetics.

[35] Fitzpatrick DR, Shirley KM, Kelso A (1999). Cutting edge: stable epigenetic inheritance of regional IFN-gamma promoter demethylation in CD44highCD8+ T lymphocytes. J Immunol.

[36] Mikovits JA, Young HA, Vertino P, Issa JP, Pitha PM, Turcoski-Corrales S (1998). Infection with human immunodeficiency virus type 1 upregulates DNA methyltransferase, resulting in de novo methylation of the gamma interferon (IFN-gamma) promoter and subsequent downregulation of IFN-gamma production. Mol Cell Biol.

[37] Tolg C, Sabha N, Cortese R, Panchal T, Ahsan A, Soliman A (2011). Uropathogenic E. coli infection provokes epigenetic downregulation of CDKN2A (p16INK4A) in uroepithelial cells. Lab Invest.

[38] Olszak T, An D, Zeissig S, Vera MP, Richter J, Franke A (2012). Microbial exposure during early life has persistent effects on natural killer T cell function. Science.

[39] Strachan DP (1989). Hay fever, hygiene, and household size. BMJ.

[40] Strachan DP (2000). Family size, infection and atopy: The first decade of the “hygiene hypothesis”. Thorax.

[41] Rook GA, Martinelli R, Brunet LR (2003). Innate immune responses to mycobacteria and the downregulation of atopic responses. Curr Opin Allergy Clin Immunol.

[42] Weiss ST (2008). Asthma in early life: is the hygiene hypothesis correct?. J Pediatr.

[43] Rook GA LCA, Raison CL (2013). Microbial ‘Old Friends’, immunoregulation and stress resilience. Evol Med Public Health.

[44] Rautava S, Ruuskanen O, Ouwehand A, Salminen S, Isolauri E (2001). The Hygiene Hypothesis of Atopic Disease—An Extended Version. Journal of Pediatric Gastroenterology and Nutrition.

[45] Blaser MJ, Falkow S. What are the consequences of the disappearing human microbiota? Nat Rev Microbiol. 2009;7:887–894. doi:10.1038/nrmicro2245. 10.1038/nrmicro2245 PMC9354563 19898491

[46] Braniste V, Al-Asmakh M, Kowa C, Anuar F, Abbaspour A, Tóth M, et al. The gut microbiota influences blood-brain barrier permeability in mice. Sci Transl Med. 2014;6(263):263ra158. 10.1126/scitranslmed.3009759 PMC4396848 25411471

[47] Koplin J, Allen K, Gurrin L, Osborne N, Tang MLK, Dharmage S (2008). Is caesarean delivery associated with sensitization to food allergens and IgE-mediated food allergy: A systematic review. Pediatr Allergy Immunol.

[48] Cardwell CR, Stene LC, Joner G, Cinek O, Svensson J, Goldacre MJ (2008). Caesarean section is associated with an increased risk of childhood-onset type 1 diabetes mellitus: a meta-analysis of observational studies. Diabetologia.

[49] Thavagnanam S, Fleming J, Bromley A, Shields MD, Cardwell CR (2008). A meta-analysis of the association between Caesarean section and childhood asthma. Clin Exp Allergy.

[50] Kendall AI (1915). The bacteria of the intestinal tract of man. Science.

[51] Madan JC, Farzan SF, Hibberd PL, Karagas MR (2012). Normal neonatal microbiome variation in relation to environmental factors, infection and allergy. Curr Opin Pediatr.

[52] Penders J, Thijs C, Vink C, Stelma FF, Snijders B, Kummeling I (2006). Factors influencing the composition of the intestinal microbiota in early infancy. Pediatrics..

[53] Smaill FM, Gyte GM, et al. Antibiotic prophylaxis versus no prophylaxis for preventing infection after cesarean section. Cochrane Database of Systematic Reviews. 2010, CD007482. doi:10.1002/14651858.CD007482.pub2. 10.1002/14651858.CD007482.pub2 PMC4007637 20091635

[54] Costantine MM (2008). Timing of perioperative antibiotics for cesarean delivery: a metaanalysis. Am J Obstet Gynecol.

[55] Ohlsson, A. and V.S. Shah, Intrapartum antibiotics for known maternal Group B streptococcal colonization. Cochrane Database of Systematic Reviews, 2014. Issue 6. Art. No.: CD007467. doi: 10.1002/14651858.CD007467.pub4. 10.1002/14651858.CD007467.pub4 24915629

[56] Verani JR, McGee L, Schrag SJ (2010). Prevention of perinatal group B streptococcal disease--revised guidelines from CDC, 2010. MMWR. Recommendations and Reports : Morbidity and Mortality Weekly report. Recommendations and Reports / Centers for Disease Control. CDC.

[57] Stensballe LG, Simonsen J, Jensen SM, Bonnelykke K, Bisgaard H. Use of Antibiotics during Pregnancy Increases the Risk of Asthma in Early Childhood. J Pediatr. 2013;162(4):832-838.e833. 10.1016/j.jpeds.2012.09.049 23140881

[58] Collier CH, Risnes K, Norwitz ER, Bracken MB, Illuzzi JL. Maternal infection in pregnancy and risk of asthma in offspring. Matern Child Health J. 2013. DOI:10.1007/s10995-013-1220-2. 10.1007/s10995-013-1220-2 23338127

[59] Metsälä J, Lundqvist A, Virta LJ, Kaila M, Gissler M, Virtanen SM (2013). Mother’s and Offspring’s Use of Antibiotics and Infant Allergy to Cow’s Milk. Epidemiology.

[60] Ajslev TA, Andersen CS, Gamborg M, Sørensen TIA, Jess T (2011). Childhood overweight after establishment of the gut microbiota: the role of delivery mode, pre-pregnancy weight and early administration of antibiotics. Int J Obes.

[61] Goto K, Yabe K, Suzuki T, Takasuna K, Jindo T, Manabe S (2008). Gene expression profiles in the articular cartilage of juvenile rats receiving the quinolone antibacterial agent ofloxacin. Toxicology..

[62] Lamouse-Smith ES, Tzeng A, Starnbach MN (2011). The intestinal flora is required to support antibody responses to systemic immunization in infant and germ free mice. PLoS One.

[63] Csoka AB, Szyf M (2009). Epigenetic side-effects of common pharmaceuticals: A potential new field in medicine and pharmacology. Med. Hypotheses.

[64] Stokholm J, Sevelsted A, Bonnelykke K, Bisgaard H (2014). Maternal propensity for infections and risk of childhood asthma: a registry-based cohort study. Lancet.

[65] Miller NM, Fisk NM, Modi N, Glover V (2005). Stress responses at birth: determinants of cord arterial cortisol and links with cortisol response in infancy. BJOG.

[66] Schlinzig T, Johansson S, Gunnar A, Ekstrom TJ, Norman M (2009). Epigenetic modulation at birth-altered DNA-methylation in white blood cells after caesarean section. Acta Paediatr.

[67] Almgren M, Schlinzig T, Gomez-Cabrero D, Gunnar A, Sundin M, Johansson S, et al. Cesarean delivery and hematopoietic stem cell epigenetics in the newborn infant: implications for future health? Am J Obstet Gynecol. 2014;1–7. 10.1016/j.ajog.2014.05.014 24996659

[68] Neumann ID (2008). Brain oxytocin: a key regulator of emotional and social behaviours in both females and males. J Neuroendocrinol.

[69] Provençal N, Suderman MJ, Guillemin C, Massart R, Ruggiero A, Wang D (2012). The signature of maternal rearing in the methylome in rhesus macaque prefrontal cortex and T cells. J Neurosci.

[70] Szyf M (2014). Epigenetic Mechanisms Embedding Social Experience in the Genome. Lowenthal Symposium.

[71] Downe S, McCourt C, Downe S (2008). From Being to Becoming: Reconstructing childbirth knowledges. Normal Birth, evidence and debate.

[72] Wright ML, Starkweather AR, York TP. Mechanisms of the Maternal Exposome and Implications for Health Outcomes. Advances in Nursing Science in press, (Accepted June 8, 2015). 10.1097/ANS.0000000000000110 PMC4860277 27149232

[73] Wright RJ, Visness CM, Calatroni A, Grayson MH, Gold DR, Sandel MT (2010). Prenatal Maternal Stress and Cord Blood Innate and Adaptive Cytokine Responses in an Inner-City Cohort. Am J Respir Crit Care Med.

[74] Peters JL, Cohen S, Staudenmayer J, Hosen J, Platts-Mills TAE, Wright RJ (2012). Prenatal negative life events increases cord blood IgE: interactions with dust mite allergen and maternal atopy. Allergy.

[75] Baron U, Floess S, Wieczorek G, Baumann K, Grützkau A, Dong J (2007). DNA demethylation in the human FOXP3 locus discriminates regulatory T cells from activated FOXP3(+) conventional T cells. Eur J Immunol..

[76] White G, Watt P, Holt B, Holt P (2002). Differential patterns of methylation of the IFN-Î3 promoter at CpG and non-CpG sites underlie differences in IFN-Î3 gene expression between human neonatal and adult. J Immunol..

[77] Liu J, Ballaney M, Al-alem U, Quan C (2008). Combined inhaled diesel exhaust particles and allergen exposure alter methylation of T helper genes and IgE production in vivo. Toxicol..

[78] Bobetsis YA, Barros SP, Lin DM, Weidman JR, Dolinoy DC, Jirtle RL (2007). Bacterial infection promotes DNA hypermethylation. J Dent Res.

[79] Schaub B, Liu J, Höppler S, Schleich I, Huehn J, Olek S, et al. Maternal farm exposure modulates neonatal immune mechanisms through regulatory T cells. J Allergy Clin Immunol. 2009;123:774–82.e5. 10.1016/j.jaci.2009.01.056 19348917

